# Isolation of a Highly Thermal Stable Lama Single Domain Antibody Specific for *Staphylococcus aureus *Enterotoxin B

**DOI:** 10.1186/1472-6750-11-86

**Published:** 2011-09-21

**Authors:** Russell R Graef, George P Anderson, Katherine A Doyle, Dan Zabetakis, Felicia N Sutton, Jinny L Liu, Joseline Serrano-González, Ellen R Goldman, Lynn A Cooper

**Affiliations:** 1 MITRE Corporation, 7515 Colshire Drive, McLean, Virginia, 22102, USA; 2 Center for Bio/Molecular Science and Engineering, Naval Research Laboratory, 4555 Overlook Avenue SW, Washington, DC, 20375, USA; 3 Department of Biology, University of Puerto Rico at Arecibo, Arecibo, 00613, Puerto Rico

## Abstract

**Background:**

Camelids and sharks possess a unique subclass of antibodies comprised of only heavy chains. The antigen binding fragments of these unique antibodies can be cloned and expressed as single domain antibodies (sdAbs). The ability of these small antigen-binding molecules to refold after heating to achieve their original structure, as well as their diminutive size, makes them attractive candidates for diagnostic assays.

**Results:**

Here we describe the isolation of an sdAb against *Staphyloccocus aureus *enterotoxin B (SEB). The clone, A3, was found to have high affinity (Kd = 75 pM) and good specificity for SEB, showing no cross reactivity to related molecules such as Staphylococcal enterotoxin A (SEA), Staphylococcal enterotoxin D (SED), and Shiga toxin. Most remarkably, this anti-SEB sdAb had an extremely high Tm of 85°C and an ability to refold after heating to 95°C. The sharp Tm determined by circular dichroism, was found to contrast with the gradual decrease observed in intrinsic fluorescence. We demonstrated the utility of this sdAb as a capture and detector molecule in Luminex based assays providing limits of detection (LODs) of at least 64 pg/mL.

**Conclusion:**

The anti-SEB sdAb A3 was found to have a high affinity and an extraordinarily high Tm and could still refold to recover activity after heat denaturation. This combination of heat resilience and strong, specific binding make this sdAb a good candidate for use in antibody-based toxin detection technologies.

## Background

The "Amerithrax" anthrax attacks of 2001 focused attention on the need for rapid and robust diagnostic methods to detect biological threat agents in environmental and clinical samples [1]. Many laboratory diagnostic platforms (eg enzyme linked immunosorbent assays [ELISAs], flow cytometry, and western blots) use target-specific antibodies to detect microbial pathogens and toxins. Antibody based assays are particularly useful for identifying highly purified biological toxins because such samples contain little if any nucleic acids on which polymerase chain reaction (PCR) assays depend [2-4]. Simplified antibody-based tests (e.g. lateral flow assays) have been developed for field analysis and are used for a wide range of applications [5,6]. However the standard reagent-grade antibodies used in these tests are heat labile, meaning that they may degrade under harsh conditions, limiting field applications [7,8]. Replacing these standard antibodies with a type of immunoreagent that is more stable could greatly simplify the logistical demands of field-deployed biosensors.

A handful of animal species produce antibodies that are functional but are devoid of light chains. These heavy chain only antibodies (HcAbs) can be isolated from members of the Camelid family and from sharks [9,10]. The variable regions of HcAb (V_HH_) when expressed as recombinant fragments, often called single domain antibodies (sdAbs), exhibit valuable characteristics including small size (12-16 kDa) and the ability to refold following heating to temperatures which normally causes the irreversible denaturation of conventional antibodies [11,12]. These properties make sdAbs attractive candidates for the development of immunodiagnostic tests [13]. Previously, sdAbs able to bind small molecules (caffeine and methotrexate), or toxins (botulinum, ricin, cholera, and scorpion), and viruses (rotavirus, HIV, Vaccinia, and Marburg) have been isolated [11,14-20]. Of particular relevance, a sdAb has recently been developed for the related toxin, toxic-shock syndrome toxin 1 (TSST-1),[21] and another for the detection of *Staphylococcus aureus *[22].

*Staphylococcus aureus *produces a number of potent enterotoxins, of which Staphylococcal enterotoxin B (SEB) is the most common cause of food borne poisoning. SEB is a single-chain polypeptide of 239 amino acids and has a molecular mass of 28.4 kDa [23]. In addition to SEB's role in food poisoning, the toxin is considered a potential biological threat agent, and is listed as a category B select agent by the Centers for Disease Control. Here we describe the isolation and characterization of an anti-SEB single domain antibody from an immunized llama, and demonstrate its utility for detecting SEB in immunoassays.

## Results and Discussion

### Evaluation of Serum and purified anti-SEB IgG

**Figure 1:**
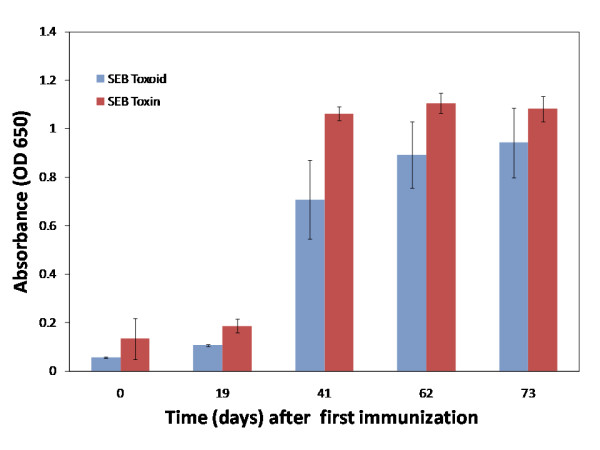
**ELISA results of direct binding of llama plasma to SEB toxin and toxoid coated wells**. This data verifies the presence of SEB toxin and toxoid binding IgG in the immunized llama plasma.

Our goal was to generate camelid sdAbs against SEB using a phage display library derived from the white blood cells of a llama serially immunized with SEB toxoid and to detail the antigen binding properties of isolated anti-SEB sdAbs. The llama (Spode) was immunized using SEB toxoid, and the presence of anti-SEB toxin antibodies in the plasma was verified by ELISA (Figure 1) prior to library construction. Once we confirmed a robust immune response towards SEB, we isolated RNA from the llama's white blood cells for library construction.

In addition to confirming the presence of anti-SEB antibodies in the llama plasma, the Immunoglobulin G (IgG) was purified and subclasses fractionated into conventional (IgG1) and heavy-chain only antibody (IgG2 and IgG3) fractions using Protein G and Protein A columns. The IgG subclasses were evaluated by fast protein liquid chromatography (FPLC). The IgG2 was clearly smaller than the IgG1 as seen by FPLC, and composed of only heavy chains as observed by polyacrylamide gel electrophoresis (PAGE) (data not shown) confirming the lack of light chains. However, the IgG3 fraction failed to separate from IgG1, thus results using this material are not shown. The lama polyclonal antibody and purified conventional (IgG1) and heavy chain only (IgG2) fractions were evaluated along with the isolated sdAb to assess their specificity and thermal stability. The llama polyclonal antibody was also paired with the isolated sdAb for use in sandwich immunoassays for the detection of SEB. These results are discussed below.

### Isolation of the anti-SEB SdAb A3

**Figure 2:**
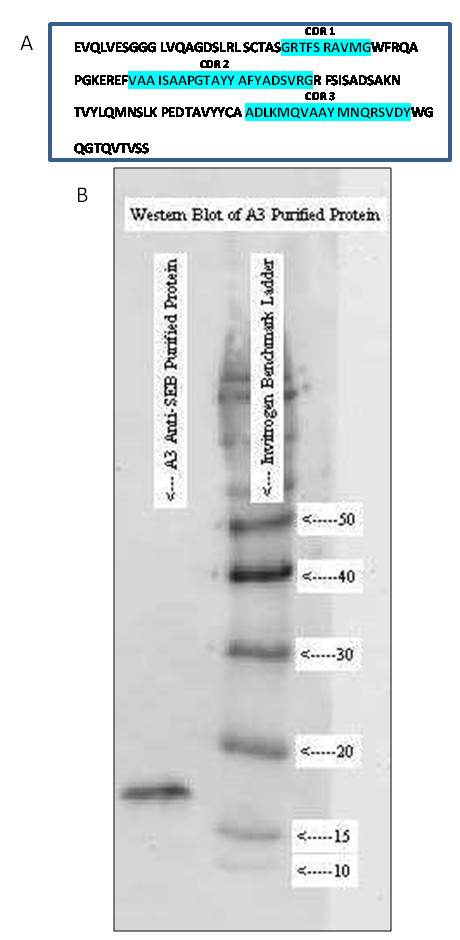
**Amino acid sequence and western blot of sdAb A3.** A) Predicted amino acid sequence of the A3 anti-SEB sdAb. CDRs are highlighted. B) Western Blot of purified sdAb A3 using anti-His-HRP antibody. Benchmark ladder.

A library was developed from the peripheral blood lymphocytes purified from the immunized llama. This library was estimated to have a size of ~10^6 ^by the number of transformants. Eleven individual clones were plaque purified and sequenced; all 11 were found to be unique and full length.  To isolate a SEB specific sdAb from the phage display library we conducted 3 rounds of panning, followed by monoclonal phage ELISA.  Following ELISA, 11 clones were selected and sequenced; all sequenced clones (n = 11) had identical base pair sequences indicating that by round 3 of panning the diversity of binders had been reduced to a single clone. The predicted amino acid sequence, represented by clone A3, is shown in Figure 2A. To evaluate its binding characteristics, clone A3 was expressed as a soluble protein and purified; its size (~16 kDa) was confirmed on a western blot (Figure 2B).

### Specificity of the anti-SEB SdAb A3 and polyclonal llama IgG

**Figure 3:**
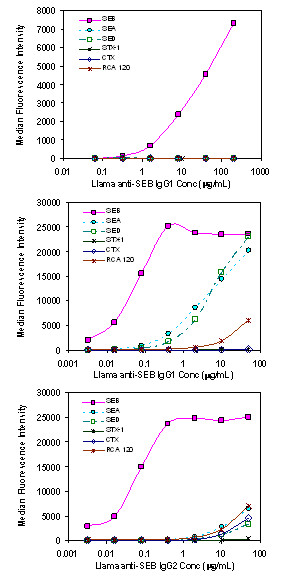
**Evaluation of specificity by Luminex fluid array direct binding assays: Bt-sdAb A3 (top panel), Bt- llama anti-SEB IgG1 (middle panel), and Bt-IgG2 (bottom panel) subclass fraction**. Result show A3 is highly specific for SEB.

The initial studies examined the cross-reactivity of A3, as well as both IgG1 and IgG2 subclass fractions, to a panel of common threat agent toxins using Luminex direct binding assays (Figure 3). We found that A3 was specific for SEB toxin and did not cross react with other toxin targets including super antigen relatives such as SEA and SED. This specificity, which is also observed in conventional monoclonal antibodies, is ideal for developing target-specific reagents for immunodiagnostics or environmental sensing. The llama polyclonal IgG1 and IgG2 subclass antibodies purified from the animal demonstrated a much higher titer for SEB relative to the other toxins, but at higher IgG concentrations they displayed cross reactivity towards SEA and SED, demonstrating the rationale for using highly selective monoclonal antibody reagents.

### Thermal stability of the anti-SEB SdAb A3 and polyclonal llama IgG

**Figure 4:**
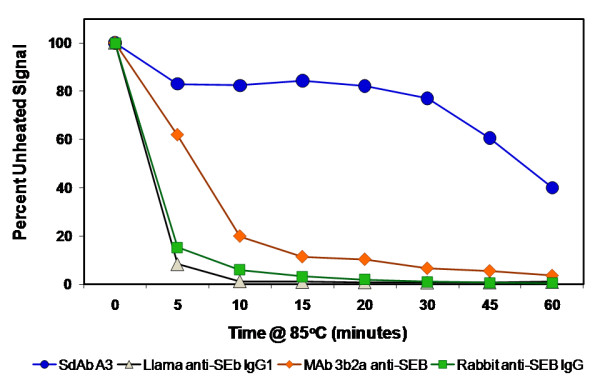
**Evaluation of thermal stability by binding activity**. SdAb A3 at 10 μg/ml and antibodies (llama anti-SEB IgG1, RA-SEB, and anti-SEB MAb 3b2a) at 100 μg/ml were heated at 85°C. Aliquots were removed and tested. The samples were diluted 10-fold and tested for their antigen binding ability in a Luminex direct binding assay.

Once the specificity of the sdAb A3 had been established we evaluated its thermal stability, comparing the selected sdAb with anti-SEB MAb 3b2a, as well as the llama polyclonal IgG1. First, an activity assay was utilized, wherein biotinylated aliquots of sdAb A3, llama polyclonal IgG, rabbit anti-SEB (RA-SEB) and anti-SEB MAb 3b2a were evaluated by heating to 85°C for various lengths of time. After cooling, their ability to bind SEB coated microspheres was evaluated (Figure 4). Within 10 minutes of heating conventional llama antibody (IgG1), as well as RA-SEB and MAb 3b2a, rapidly lost 80% of their binding activity and after an hour they had lost over 95%. The binding activity of the llama heavy chain antibody subclass IgG2 initially declined 40% during the first 5 minutes of heating but then declined only slowly over the remaining forty five minutes, retaining ~30% of the initial activity. Clearly, the sdAb A3 exhibited the best stability, decreasing slightly (20%) upon initial heating but then residual activity remained fairly constant, dropping again during the last 15 minutes to 45% of the initial activity after heating for 1 hour.

**Figure 5:**
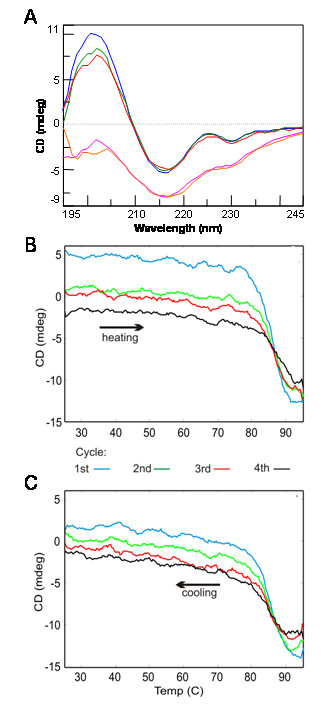
**Evaluation of A3's thermal stability by monitoring secondary structure by CD**. A3 was heated to 85°C and then cooled to 25°C repeatedly: A) CD spectra of A3 (blue line) and upon heating to 85°C (1^st ^cycle, pink; 2^nd ^cycle, orange) and upon cooling to 25°C (1^st ^cycle, green line; 2^nd ^cycle, red line). Alternatively, secondary structure can be monitored and Tm determined by tracking the ellipticity change at single wavelength 202 nm: B) 4 heat cycles to 95°C, first cycle blue, second cycle green, third cycle red, and fourth cycle black; C) 4 cooling cycles color coded as in panel B.

**Figure 6:**
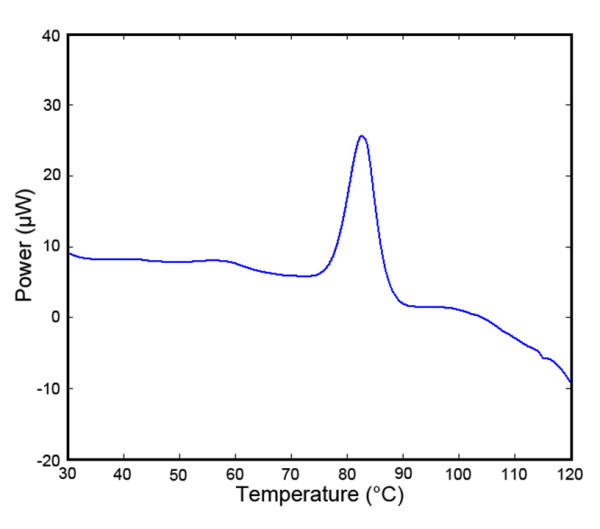
**Differential Scanning Calorimetry of the sdAb A3**. Antibody melting was observed in PBS with a protein concentration of 1.6 mg/ml. Heating rate was 1°C per minute. Melting temperature was about 82.6°C and refolding upon cooling was not observed.

**Figure 7:**
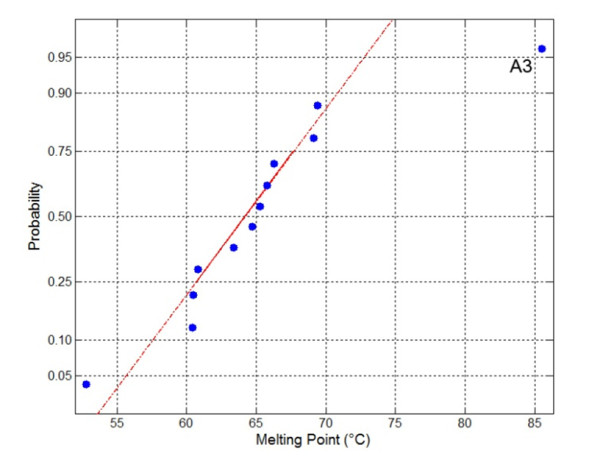
**The Tm of A3 deviates from a normal distribution**. A total of 12 SdAb for which the melting temperatures have been determined are presented on a normal probability plot. The Y-axis values are specified by the probability distribution function for an assumed normal distribution. The straight line is a robust linear regression of the data and all data points will be near the line if the assumption is correct. The Tm for A3 can be seen to fit poorly.

Next, we examined the effect of heating on the secondary structure of the antibodies (non-biotinylated) by monitoring their CD spectrum during heating. The CD spectrum shows that the sdAb A3 unfolded upon heating and then refolded when cooled (Figure 5A). A3 was then subjected to multiple cycles of heating and cooling (Figure 5B and 5C). By monitoring the ellipticity at 202 nm, A3 was observed to start losing secondary structure at 80°C, with a melting point at ~85°C. After cooling down, the sdAb was found to recover the majority of its secondary structure. The unfolding and refolding transitions occurred rapidly and at nearly the same temperature. Clone A3 has one of the highest melting points we have observed to date for a sdAb [24-29]. To confirm this melting point, this value was also determined using DSC (Figure 6). This method yielded a melting point of 85°C, however under these conditions, high concentration and high maximum temperature, no refolding was observed. To evaluate the melting temperatures of sdAbs, including A3, we constructed a normal probability plot (Figure 7). Points on this plot will lie along the regression line if the data conforms to a normal distribution. For 11 antibodies for which Tm has been determined (unpublished observations) the data can be seen to fit well to a normal distribution. A3, with a Tm above 85°C, can be seen to deviate significantly, implying that this data cannot be explained as an expected variation in a normal distribution.

**Figure 8:**
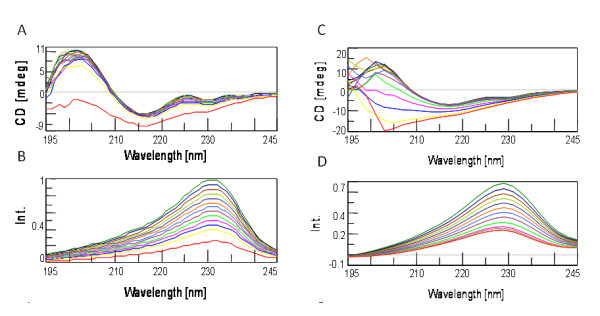
**Comparison of CD spectra and intrinsic fluorescence intensity of sdAb A3 to llama IgG1 taken at 5°C intervals from 25°C to 85°C**. SdAb A3 a) CD spectra b) fluorescence intensity. Llama anti SEB IgG1 c) CD spectra d) fluorescence intensity. Results indicate that intrinsic fluorescence changes fail to correlate with the sdAb's loss of ellipticity upon heating, while intrinsic fluorescence does correlate with ellipticity for the polyclonal IgG.

Another interesting finding was that even though the sdAb A3 maintains its secondary structure until 80°C, its intrinsic fluorescence decreased in a nearly linear relationship with temperature (Figure 8A and 8B). The decrease in fluorescence intensity can be attributed to the decreasing hydrophobic environment of buried tryptophans as well as to increased vibrational decay due to increased motion. Fundamentally, these results indicate that for sdAb, fluorescence intensity is a poor metric to monitor protein folding, with the actual CD spectra providing a more precise Tm. These results are contrasted by the CD and fluorescence measurements of the llama IgG1 anti-SEB, which lost both secondary structure and fluorescence intensity at comparable rates (Figure 8C and 8D). More importantly, conventional IgGs do not refold properly after heating, permanently losing the bulk of their secondary structure and activity. The thermal resilience shown by the sdAb could be extremely useful for detection systems because sdAb molecules could be repeatedly used to capture their target simply by serially heating and cooling. Such an application would be an important contribution to current bio-detection technologies as it could greatly enhance the ability to field continuous monitoring systems.

### Affinity of the anti-SEB sdAb A3

**Figure 9:**
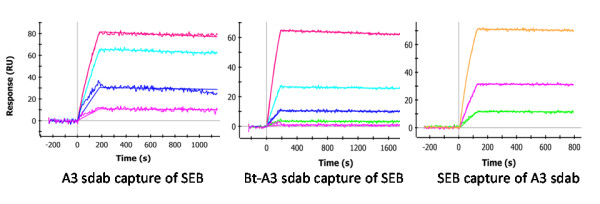
**SPR binding profiles for sdAb A3**. Panel A shows five concentrations of SEB (30, 10, 3.3, 1.1, and 0.37 nM) flowed for 3 minutes over immobilized sdAb A3, followed by 15 minutes dissociaiton. Data shown was corrected by subtraction of interspot data and the buffer only response. Panel B shows a similar experiment performed on a NeutrAvidin coated chip with Bt-sdAb A3 immobilized. Panel C shows sdAb A3 (30, 10, 3.3, 1.1, and 0.37 nm binding to a surface coated with SEB.

The affinity constants of sdAb A3 were determined using SPR (Figure 9). A3 was found to have a very high affinity for SEB, Table 1. Similar results were obtained from both the covalently immobilized A3 and the Bt-A3 bound to a NeutrAvidin coated chip, with both yielding a KD of ~ 400 to 600 pM. When the SEB was immobilized, A3 bound even more effectively, with a measured KD of ~ 75 pM, confirming the high affinity interaction that A3 has for SEB. The MAb 3b2a also had a very high affinity for SEB. In fact it's off rate was so low we were unable to determine an accurate affinity (KD ≤ 100 pM). These high affinities explain the ability of these immunoreagent to sensitively detect SEB as demonstrated below.

**Table 1:** Result of SPR binding analysis

Capture Molecule	ka 1/Ms	kd 1/s	KD
SdAb-A3	1.0 × 10^+5^	6.0 × 10^-5^	6.0 × 10^-10^

NA-Bt-A3	7.2 × 10^+4^	2.8 × 10^-5^	3.9 × 10^-10^

SEB	2.9 × 10^+5^	2.2 × 10^-5^	7.5 × 10^-11^

The kinetic binding results obtained from the data shown in Figure 8. Kinetic parameters were determined by fitting the curves using a Langmuir model.

### Sandwich immunoassays for the detection of SEB using the anti-SEB sdAb A3

**Figure 10:**
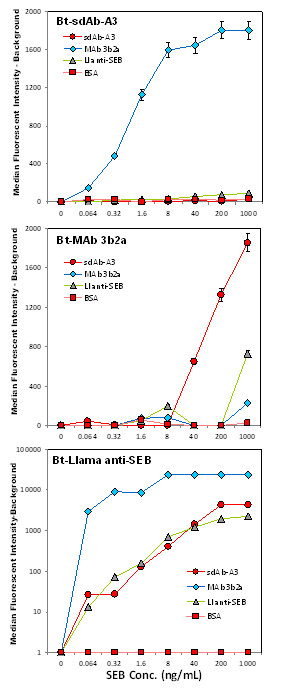
**SEB detection using Luminex sandwich assays**. Three different anti-SEB antibody coated bead sets plus a negative control were used in each assay performed as described in the methods. Top panel used Bt-sdAb-A3 (1 μg/mL), middle panel used Bt-MAb 3b2a (10 μg/mL), and bottom panel Bt-Llama anti-SEB (10 μg/mL), respectively as the detector antibody, followed by SA-PE (5 μg/mL) to fluorescently label the immuno-complex prior to measurement on the Luminex 100.

The final studies utilized the sdAb A3 as either the capture molecule or the detection molecule in fluid array sandwich immunoassays for SEB. In addition to A3, the MAb 3b2a and the purified llama polyclonal anti-SEB were utilized (Figure 10). SdAb A3 functioned well when paired with MAb 3b2a in the sandwich immunoassay indicating that they recognize different epitopes. When A3 was the Bt detector and MAb 3b2a the capture molecule (Figure 10 top panel) the assay could detect as little as 64 pg/mL SEB. The reverse configuration, Bt-MA3b2a detector and sdAb A3 capture (Figure 10 middle panel), was not as sensitive with a LOD of 40 ng/ml. While the llama polyclonal antibody was a poor capture molecule, presumably due to its relatively low titer, the Bt-llama polyclonal antibody functioned very effectively as a Bt-labeled detector in combination with all the captures (Figure 10 bottom panel). Limits of detection of 64 pg/mL SEB were observed with the sdAb A3 and llama IgG1 anti-SEB captures, while when paired with the monoclonal it gave a signal intensity ~10-fold greater, suggesting that pair would have an even more impressive LOD. The large differential between the A3 and the Mab 3b2a capture molecules when using the polyclonal detector may occur because the polyclonal antibody favors the same epitope as the A3. Nonetheless, the A3 sdAb provided detection limits to 64 pg/mL as both a capture and detector molecule, which is more than adequate to meet most current detection requirements. In addition, the thermal stability and ability to re-fold after denaturation should give sdAbs, such as A3, an advantage when incorporated into biosensors for use in austere environments. For such applications it would be advantageous to isolate a second anti-SEB sdAb that recognized a different epitope towards developing a sandwich immunoassay not reliant on monoclonal or polyclonal conventional antibodies. The selection of such sdAb from this same library for the development of an all sdAb immunoassay is the next goal of this research effort.

## Conclusions

In conclusion, the antigen recognition capabilities of the llama derived sdAb A3 were strong and specific for its target, SEB. The A3 sdAb was also found to be extremely heat resilient and to refold correctly following heat denaturation. These traits make it a valuable contribution to the current biodefense and public health arsenals of threat detection molecules. The availability of high affinity and recyclable recognition elements, such as A3, might also be used to increase the functional range of biosensors in the near future.

## Methods

### Immunization Protocol and Serology

An adult male llama housed at Triple J Farms (Bellingham, WA) was immunized with purified SEB toxoid (Toxin Technologies) intramuscularly followed by three boosters at three week intervals with 225 μg of purified SEB toxoid. Sera was collected immediately before each inoculation and used to monitor the total IgG response against SEB. Three weeks after the final immunization 500 mL of whole blood and 50 mL of plasma was collected and used for lymphocyte isolation and serology. White blood cells were immediately isolated from the whole blood for RNA purification. Plasma was stored at -80°C until used. Animal studies were approved by a separate review board through Triple J Farms and Kent Laboratories.

Total IgG responses to SEB toxin (Sigma Aldrich) and toxoid were measured over the course of the immunization series by direct ELISA. Briefly, plate wells were coated with 0.5 μg/mL of SEB toxin or toxoid overnight at 4°C. The following morning wells were washed three times with phosphate buffered saline (PBS) followed by addition of 3% non-fat Milk in PBS (MPBS) and kept at 37°C for two hours. Afterward, wells were washed again three times with PBS followed by coating them with diluted sera (1:1000) for 1 hour at 37°C. The wells were again washed three times with PBS and incubated with anti-llama IgG (H+L) horse radish peroxidase (HRP) Conjugated (Bethyl Laboratories) at 1:5000 for 1 hour. Finally, wells were washed three times with PBS before the addition of substrate reagent pack (R and D Systems), and signal was read by optical density (OD) at 455 and 650 nm.

### IgG antibody purification

IgG was purified from llama plasma using a two-step process of Caprylic Acid (CA) to partially purify the IgG followed by using MEP HyperCel hydrophobic charge induction chromatography as previously described [30,31]. The IgG was separated into subclasses by a combination of affinity chromatography on protein G and protein A columns as described previously [32-34].

### Phagemid Library Construction and Panning

White blood cells were isolated from the final post immunization bleed using Ficoll-Plaque Plus (GE Lifesciences), pelleted and washed with PBS, pelleted and stored at negative 80°C in Trizol (Invitrogen) until used for RNA extraction. RNA was extracted from Trizol and converted to cDNA using Arrayscript (Ambion) via oligo-dT primed reverse transcription. V_HH _coding regions were amplified by nested PCR as previously described [32,35]. The first round, 600 bp amplicon, was agarose gel purified using Ultrafree-DNA kit (Millipore) and used as the template for the secondary PCR reaction using the nested primers which amplify a 450 bp region and incorporate SfiI and NotI restriction sites into the 5' and 3' ends, respectively [15]. The 450 bp amplicons were agarose gel purified as before and then digested with SfiI and NotI. The resulting fragments were gel purified using Ultrafree-DNA system and concentrated with the Microcon YM-100 (Millipore). Purified DNA was ligated, using T4 DNA Ligase, into an open M13 phagemid vector, pCANTAB 5 E (GE Lifesciences). The ligations were then desalted and electroporated into electrocompetent XL-1 Blue *Escherichia coli *(Stratagene) and then pooled to form the library. A 10-fold serial dilution series of the library was plated out on Luria Broth plates containing 100 μg/mL Ampicillin (LB amp100) and colony counts used to estimate total library size of ~10^6 ^variants. Phage displayed SEB-specific V_HH _antibodies were isolated by phage rescue and panning. Phages were rescued via M13K07 phagemid purification [36]. Screened isolates were then used as the starting point for the second round of panning, and this process was repeated twice more for a total of 3 rounds of selective panning. Third round isolates were plaque assayed, randomly chosen from plates, to be screened for SEB-specific binding by ELISA. The plate was coated overnight with 5 μg/mL SEB in PBS. The wells were then blocked with 3% MPBS for 2 hrs at 37°C. PEG precipitated phage from 96 individual colonies was added to each well, and the plate was incubated for 90 minutes at room temperature. Anti-M13-HRP conjugate (GE Lifesciences) diluted 1:5000 in MPBS was added to each well and incubated for 90 min at room temperature. Plates were developed with the substrate reagent pack, and the reaction was monitored at OD 455 and 650 nm. Clones were ranked based on absorbance and the top 11 chosen for further characterization.

### Sequencing

DNA sequences spanning the V_HH _portion of each of the 11 high binders were determined by commercial sequencing provided by MCLAB. Sequences were amplified using lacZ' forward primer 5'-ctatgaccatgattacgaatttctag-3'. Sequence analysis was performed using Geneious software (Biomatters Ltd.).

### Protein Expression and Purification

A single sdAb variant, A3, was chosen for further analysis and cloned into the pecan45 expression vector (kindly provided by Andrew Hayhurst, Southwest Foundation for Biomedical Research) and transformed into BL-21 Rosetta *Escherichia coli *cells (Novagen). SdAb protein was overexpressed and purified following protocols described previously [11]. In brief, A3 was overexpressed using Isopropyl β-D-1-thiogalactopyranoside (Fisherbrand); proteins were extracted from the periplasmic compartment by osmotic shock and purified by Immobilized Metal Ion Affinity Chromatography (IMAC) via incubation and elution from Nickel Sepharose (GE Lifesciences) [37]. Purified protein was stored at 4°C prior to gel filtration on a Superdex G75 column (GE Lifesciences) [11]. Protein was quantified using micro-BCA assay (Pierce) following manufacturer's protocols.

Western blots were preformed to confirm the purified protein's size. Purified protein samples were analyzed by SDS-PAGE on 12% Tris-Cl polyacrylamide gel by electrophoresis for 37 minutes at 200 V. Following electrophoresis the gel was transferred to a nitrocellulose membrane (BioRad) for 1 hour at 100 V. Membranes were incubated overnight at 4°C with MPBS with 0.05% Tween 20 (MPBST), followed by incubation with anti-6 × His-HRP antibody (GenScript) for 1 hour at room temperature with shaking. The membranes were developed using Immuno-star Western C kits (Bio-Rad) following the manufacture's protocols, and images were captured on Versadoc (BioRad).

### Luminex Analysis

A Luminex 100 flow analyzer was used to determine the specificity of the isolated sdAb as well as to perform sandwich assays for SEB. Antibodies were biotinylated (Bt) as previously described [25] using NHS-LC-biotin dissolved first in DMSO; a 20:1 NHS-biotin molar excess was used. Gel filtration on a Bio-gel P10 column was used to separate the biotin excess. Luminex microspheres for direct binding assays were coated with: two lots of SEB toxin, bovine albumin (BSA) (Sigma); Shiga Toxin 1 (STX-1), staphylococcal enterotoxin D (SED), staphylococcal enterotoxin A (SEA) (Toxin Technologies); Ricin, *Ricinus communis *Agglutinin 120 (RCA-120) (Vector); Cholera Toxin (CTX) (Calbiochem). For sandwich immunoassays, microspheres were coated with the A3 sdAb, the purified polyclonal llama anti-SEB, and a monoclonal anti SEB MAb 3b2a kindly provided by Dr. Jill Czarnecki (Naval Medical Research Center, Silver Spring, MD). The protocol provided by Luminex for two-step carbodiimide coupling as previously described [11] was utilized to coat 0.1 mL of microspheres using 0.1 mg protein at a concentration of ~1 mg/mL. The protein-coated microspheres were stored at 4°C in the dark until use.

Sandwich immunoassays for SEB were performed using A3 as both the capture molecule as well as the biotinylated detector In addition, the monoclonal antibody MAb 3b2a and the purified llama polyclonal anti-SEB (Ll-anti-SEB) antibody were also tested as the capture and detector molecule. SEB at a variety of concentrations was incubated with the antibody coated microspheres for 30 minutes. Unbound SEB was removed by filtration and the microspheres washed twice with PBS containing 0.05% Tween-20 (PBST). The microspheres were then incubated with biotinylated detector antibodies: Bt-A3 (1 or 2 μg/mL), Bt-Mab 3b2a (5 μg/mL), Bt-Ll-anti-SEB (10 μg/mL) for 30 minutes. After washing twice the amount bound was fluorescently labeled by the addition of 5 μg/mL SA-PE. After 30 minutes the excess SA-PE was removed and the microspheres resuspended in 85 μL PBST and measured on a Luminex 100.

Thermal stability was tested using biotinylated antibodies (polyclonal llama anti-SEB IgG, MAb 3b2a, and sdAb A3 anti-SEB). The antibody samples were heated in a thermocycler at 85°C for different times up to 60 minutes, stored at 4°C for 1 hour, and then evaluated using a direct binding Luminex assay. Specificity of binding of the A3 variant was evaluated by a cross-reaction panel to various toxins and proteins (see above list) coupled to microspheres via a direct binding assay. Purified A3 sdAb was allowed to bind for 30 minutes at room temperature then the entire panel was run simultaneously on the Luminex.

### Circular Dichroism (CD)

The melting point of the A3 sdAb was measured by circular dichroism using a Jasco J-815 CD spectropolarimeter equipped with a PTC-423S single position peltier temperature control system. Samples (~30 μg/mL) were prepared by extensive dialysis versus 5 mM sodium borate pH 7.5, or by simple dilution with that same buffer. All measurements were made in a 10 mm path length quartz cuvette with a stir bar. The data were acquired from 245 nm to 195 nm at a scanning speed of 20 nm/min. The data pitch was 1 nm, D.I.T. 2 seconds, band width 1 nm, temperature ramp rate of 5°C/min over the range of 25 to 95°C. Intrinsic protein fluorescence intensity was also measured simultaneously using a 345 nm filter.

### Differential Scanning Calorimetry (DSC)

The melting point was also measured by DSC using a TA Instruments NanoDSC. Antibody at a concentration of 1.6 mg/ml in PBS was scanned at a rate of 1°C per minute from 25°C to 120°C.

### Surface Plasmon resonance (SPR) kinetics analysis

The SPR kinetic measurements were performed using the ProteON (Bio-Rad). For testing the kinetics of the anti-SEB sdAb A3 a variety of surfaces were prepared. In one case a GLC chip was coated with the sdAb A3 (5 μg/mL) along with sdAb specific for other targets (none of which bound SEB and served as negative controls for the tests presented here). A GLC chip was also coated with SEB (5 μg/mL), as well as other toxins which served as negative controls. All the protein samples to be immobilized were diluted in 10 mM acetate buffer pH 5.0 following the standard EDC coupling chemistry provided by the manufacturer. To ensure that biotinylation of the sdAb A3 had no affect on its activity, a NeutrAvidin coated chip was coated with Bt-sdAb A3. All experiments were performed at 25°C. The binding of the sdAb were tested by flowing 6 concentrations varying from 30 nM to 0 nM for 180 seconds over the SEB coated chip, and then monitoring dissociation for 900 seconds. The chip was regenerated using 50 mM glycine-HCl (pH 2.0) for 36 seconds, prior to any additional testing. The data were analyzed with the ProteON Manager TM 2.1 software; binding constants were determined using the software's Langmuir model. Assays for binding of SEB by the immobilized sdAb were done in an analogous manner.

## Competing interests

The authors declare that they have no competing interests.

## Authors' contributions

RRG developed the phage display library, selected the A3 binder, and produced the soluble protein and wrote the manuscript. GPA performed the SPR and Luminex 100 immunoassay testing and edited the manuscript. KAD titered the llama immune serum. DZ performed most of the CD and DSC experiments and evaluated TM to develop Figure 7. JLL assisted with the library development. JSG purified the IgG subclasses and tested the thermal stability by CD and fluorescence and Luminex 100. ERG devised experiments, managed the NRL efforts and edited the manuscript. LAC conceived of this project, managed the efforts at MITRE, and edited the manuscript. All authors read and approved the final manuscript.
